# Anion Exchange Chromatography to Determine mRNA Encapsulation in Lipid Nanoparticles

**DOI:** 10.1021/acs.analchem.5c03299

**Published:** 2025-08-22

**Authors:** Athanasios Tsalmpouris, Sofiane Mahjoubi, Camille Malburet, Chamsan Daher-Hassan, Marc François-Heude, Jean-François Cotte, Davy Guillarme, Jonathan Maurer

**Affiliations:** † School of Pharmaceutical Sciences, 27212 University of Geneva, CMU-Rue Michel Servet 1, 1211 Geneva, Switzerland; ‡ Institute of Pharmaceutical Sciences of Western Switzerland, 27212 University of Geneva, CMU-Rue Michel Servet 1, 1211 Geneva, Switzerland; § mRNA Center of Excellence, Analytical Sciences, Sanofi, 1541 Avenue Marcel Mérieux, 69280 Marcy l’Etoile, France

## Abstract

Encapsulation efficiency (EE) of mRNA-based therapeutics and vaccines is defined as the percentage of total mRNA that is efficiently protected by the delivery vehicle from nuclease degradation. As a critical quality attribute, EE must be assessed to ensure that sufficient mRNA evades enzymatic degradation and traverses biological barriers to reach the cellular machinery for translation, without triggering unwanted immune responses caused by free mRNA. In this study, we developed a strategy based on anion exchange chromatography (AEX) to separate lipid nanoparticles (LNPs) and free mRNA based on their charge differences. Carryover issues were mitigated by using a washing step with surfactant, high pH, and high salt concentration. EE was determined by analyzing undiluted samples for free mRNA and measuring total mRNA after LNP disruption using surfactants. The method was successfully applied to the analysis of 30 different mRNA-LNP samples to determine EE. The results were compared to those obtained with the RiboGreen assay, one of the reference methods to assess EE. Our results revealed significant discrepancies between the two techniques that could be explained by the structural information obtained under AEX conditions. Indeed, while the RiboGreen assay provides information on the quantification of mRNA accessible to the fluorescent dye, AEX allows relative quantification of mRNA dissociated from LNPs and information on the presence of surface-localized mRNA as well as transmembrane mRNA. These findings establish AEX as a reliable EE assay, providing information on mRNA distribution within LNPs and advancing the fundamental understanding of LNP structure–function relationships. Based on these features, it offers critical guidance for the rational design of next-generation mRNA therapeutics.

## Graphical abstract

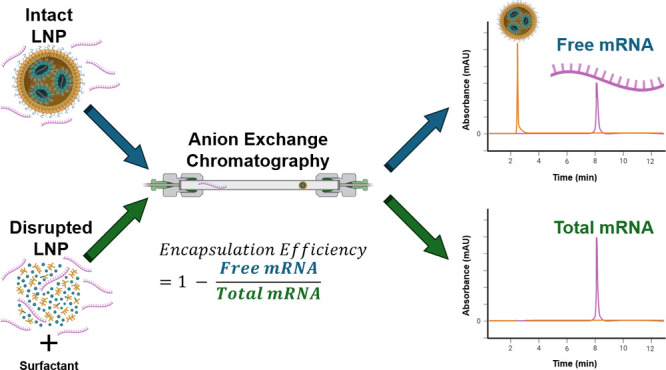

## Introduction

In recent years, there has been a growing demand for new modality therapies utilizing mRNA (mRNA) as a drug substance, particularly since the COVID-19 pandemic era. The development of in vitro transcription (IVT) has supported the practical use of mRNA, by facilitating high-yielding, large-scale synthesis strategies.^1,2^ Indeed, IVT of mRNA allows large-scale, cell-free production of mRNA and ensures the generation of high-quality transcripts, making mRNA an attractive candidate for drug development.^3–5^ On the other hand, the molecule itself is prone to degradation from RNases upon entering the human body and its large size and negative charge inhibit efficient cellular uptake.^6^ To address these challenges, lipid nanoparticles (LNPs) have emerged as a powerful RNA delivery system, capable to transport intact mRNA from the administration site into the cytoplasm, where translation can occur.^7–9^ LNPs are typically composed of four essential components: an ionizable lipid, a phospholipid, cholesterol, and PEG-lipid conjugates.^10,11^ Each of these lipids plays a crucial role in efficient intracellular delivery. Recent advances in microfluidic synthesis technologies have further enhanced the production of LNP-mRNA formulations by enabling precise control of particle size, composition, and reproducibility, which are key attributes for scalable and rapid manufacturing.

Defining the critical quality attributes (CQAs) of mRNA and LNPs remains an evolving challenge in the development of this new modality. The US Pharmacopeia, European Pharmacopeia Commission, and World Health Organization have published guidelines outlining several relevant CQAs; however, specific acceptance criteria have not yet been formally established. Yan et al. provided a comprehensive overview of CQAs for mRNA throughout product development and manufacturing and proposed additional CQAs along with suitable analytical procedures.^12^ One of the key elements in the quality control of mRNA-LNP products has been the encapsulation efficiency (EE) of the drug substance in the LNP. EE refers to the proportion of mRNA effectively enclosed within the LNPs, a process that relies on electrostatic interactions between the positively charged ionizable lipid and the negatively charged mRNA. The LNP structure protects the mRNA, which can then be safely delivered to the cell for translation and production of the intended protein.^13^ This protective envelope ensures efficiency of the administered dose and reduces unwanted immune response due to free mRNA.^14^ More than a simple measure of mRNA quantity inside the LNP, encapsulation efficiency defines the proportion of mRNA that will eventually be internalized by the cells. Therefore, it is directly linked to the structure of the mRNA-LNP complex and the protection it provides against RNases, rather than the mRNA quantity inside an LNP. Importantly, mRNA located on the surface or partially embedded within the LNP membrane may not be considered fully encapsulated, but the LNP can still offer sufficient protection against RNase to enable successful target delivery.^15,16^ Consequently, robust analytical methods for EE should ideally provide information related to the spatial distribution and localization of the mRNA species within or around the LNP.

Current methods for measuring EE often involve fluorescent dyes that bind mRNA and can then be detected by spectrofluorimetry.^17,18^ The most widely used dye is RiboGreen, a product from Thermo Fisher Scientific.^19–21^ Although RiboGreen is very sensitive (LOQs of about 100 pg RNA), it also has several limitations.^22^ The main drawback of the RiboGreen assay is its susceptibility to matrix effects. Indeed, fluorescence depends on the ability of the fluorescent dye to bind to the mRNA, and this interaction is highly sensitive to environmental factors such as ionic strength, pH, and buffer composition.^12,23,24^ Moreover, the RiboGreen assay is strongly affected by dilution, which can change the LNP structure and permeability, eventually modifying the accessibility of mRNA to the dye. As a result, it may reflect changes in LNP structure or membrane properties rather than accurately measuring how much mRNA is encapsulated.^25^ Another issue is that RiboGreen measures fluorescence only and not the real concentration of mRNA, so the results can sometimes be unreliable and hard to reproduce. Another limitation is its narrow dynamic range. While the technique works well at low mRNA concentrations, it becomes inaccurate at high concentrations due to the optical saturation effect.^26^ Lastly, the RiboGreen assay is a long and tedious process and uses a costly reagent, which makes it less practical for routine applications.^27,28^ Because of all of these challenges, there is a clear need for alternative techniques to reliably measure EE.

Several chromatographic techniques have been developed to characterize mRNA.^29,30^ The most widely used is ion-pairing reversed-phase chromatography (IP-RPLC), which uses charged tertiary amines to improve the retention of mRNA on a hydrophobic stationary phase.^31,32^ However, when analyzing mRNA encapsulated in LNPs, the sample first needs to be deformulated. Alternatively, Imiołek et al. recently considered size exclusion chromatography (SEC) to perform online disruption of LNPs and further evaluation of cargo integrity.^32^ This technique effectively liberates the mRNA cargo and assesses its integrity as it uses moderate heat, organic solvent, and surfactant as mobile phase additives, which are known to induce LNP disruption. However, both IP-RPLC and SEC are hardly compatible with the analysis of intact LNPs needed for the evaluation of free mRNA. More recently, Maurer et al. suggested that hydrophilic interaction chromatography (HILIC) holds promise for EE evaluation by using non-disruptive conditions that maintain LNP structure.^33^ The authors acknowledged that this proof-of-concept approach needs further exploration, validation, and comparison with orthogonal techniques to confirm its reliability.

Although online disruption of LNPs is easily achieved, developing chromatographic conditions that preserve LNP integrity is much more challenging. In this context, anion exchange chromatography (AEX) appears as a promising technique for EE evaluation. In AEX, positively charged compounds are retained and eluted with a salt gradient. Moreover, this approach is performed at room temperature and without organic solvents, offering conditions compatible with poorly stable LNPs.^34^ In addition, the column does not retain neutral molecules, such as intact LNPs and other uncharged impurities, resulting in clear separation of the species. By preserving the intact LNP structure, AEX emerges as a promising alternative to the RiboGreen assay and to other chromatographic techniques that risk LNP disruption. Hengelbrock et al. demonstrated the potential of AEX by using a TSKgel DNA-NPR column to monitor EE during continuous production of mRNA.^35^ Similarly, Hara et al. used a DNAPac PA200 column and a Na_2_HPO_4_/NaClO_4_ gradient to separate the poly-A tail from LNPs.^36^ Although these methods showed promising EE data, they did not capture the complexity and heterogeneity inherent to industrial R&D samples. Full-length mRNA-LNP drug products, as studied here, present a greater analytical challenge compared to synthesized poly-A tail samples because of secondary and tertiary structures, sequence heterogeneity, and different LNPs formulations. The challenges faced during method development highlight the need to work with realistic samples.

To address this need, we successfully developed an AEX method using a nonporous stationary phase to evaluate EE in mRNA-LNP formulations. The method was initially optimized for mRNA drug substances and subsequently applied to various drug products. Disruption of the LNP structure was achieved by incorporating surfactants in carefully optimized buffer systems, allowing for effective release and analysis of the mRNA cargo.

During method development, we observed the presence of large particles in our samples that caused light scattering. Our analysis suggests these particles are LNPs with mRNA bound to their surface, which results in retention in the AEX stationary phase. The occurrence of these species varied across samples and increased with extended storage at room temperature, suggesting not only structural differences between samples but also a progressive change in LNP structure over time. By implementing an isocratic step in the gradient method, we successfully separated these species from the main mRNA peak and accurately quantified both free and total mRNA. The method was subsequently applied to actual mRNA-LNP therapeutics and vaccine candidates under development, and results were compared to those obtained using the RiboGreen assay. To our knowledge, this is the first time a thorough investigation of mRNA carryover and mRNA–LNP interactions has been conducted on real drug products. Using samples that reflect industrial requirements is of great importance, since our results were highly influenced by drug-product formulation and storage conditions.

## Experimental Section

### Chemicals and Samples

Ultrapure water was obtained from a Milli-Q purification system from Millipore (Bedford, MA, USA). RNase-free water, Triton X-100, and reduced Triton X-100 were purchased from Sigma-Aldrich (Buchs, Switzerland). LC-MS grade acetonitrile and Tris-EDTA RNase-free Buffer (TE20X) were purchased from Thermo Fischer Scientific (Reinach, Switzerland). TE20X is a concentrated stock solution commonly used in molecular biology, composed of Tris-HCl (200 mM, pH 7.5–8.0) and EDTA (4 mM).

The method was developed using samples provided by Sanofi (Marcy-l’Etoile, France). In this study, drug substance samples (DS) containing mRNA in aqueous solution were used at a concentration of 1 mg/mL and drug product samples (DP) containing LNP-encapsulated mRNA at concentrations ranging from 0.26 to 2 mg/mL. Briefly, the mRNA-LNP production process combines two distinct phases using a T-mix setup: an aqueous phase containing mRNA in acidic buffer (≈pH 4) and an ethanol phase containing a mixture of four lipids (ionizable lipid, DOPE, cholesterol, DMG-PEG). The resulting LNPs undergo concentration, purification, and buffer exchange through tangential flow filtration. The final preparation includes clarification and sterilization via 0.22 μm filtration, followed by storage at −80 °C.

### HPLC Instrumentation, Columns, and Experimental Conditions

AEX analyses were performed using a Waters ACQUITY UPLC H-class System (Waters, Milford, MA, USA), equipped with a 15 μL flow-through needle injector, a quaternary solvent manager equipped with a 250 μL mixing chamber, and a UV photodiode array detector. UV signals were monitored at wavelengths of 230 and 260 nm. Data acquisition and instrument control were performed by Empower 3 software from Waters.

Analyses were performed on two different columns that were compared in terms of robustness, propensity to carryover, and overall suitability for the application. The ProPac 3R SAX 4 mm × 100 mm, 3 μm from Thermo Fischer Scientific (Reinach, Switzerland) was compared to the Accura BioPro IEX QF 4.6 mm × 100 mm, 3 μm from YMC Europe (Dinslaken, Germany).

For the final method, mobile phase A (MPA) was composed of 25 mM glycine, pH 10.1; mobile phase B (MPB) contained glycine 25 mM and NaCl 1.5 M, pH 10.1; and mobile phase C (MPC) was composed of 25 mM glycine, NaCl 3 M, and Triton X-100 reduced 0.05% v/v, pH 11.0. The AEX mobile phases were systematically filtered through a 0.22 μm PVDF membrane filter (Durapore, Merck Millipore) prior to use.

The RiboGreen assay was performed as described by Malburet and collaborators.^37^ Briefly, fluorescence measurements were performed by using a SpectraMax I3X microplate reader with excitation at 485 nm and emission at 530 nm. A standard curve (0.2–1.8 μg/mL) was prepared using reference mRNA stored at ≤−20 °C. Samples were serially diluted, with three independent preparations per sample. RiboGreen working solution was freshly prepared at a 1:200 dilution in assay buffer (TE 10X). Measurements were performed in triplicate in 96-well black plates. Free mRNA was first measured, followed by total mRNA quantification after LNP disruption by using Triton X-100. An internal control was included in each analytical series to monitor method stability.

### Qualification Data

The linearity of the responses observed with the two columns was compared using the *R* ^2^, slope, and *y*-intercept measured on a DS sample diluted in TE20x at the following concentrations 0.200, 0.100, 0.075, 0.050, 0.025, and 0.010 mg/mL, in three independent runs.

For the evaluation of accuracy, we developed an approach where the drug products were spiked with different concentrations of the DS sample diluted in TE20x. The concentrations were calculated to represent 5, 10, 20, 40, and 50% dilution. This approach allowed evaluation of the impact of the matrix, specifically the proportion of mRNA that interacts with the LNPs in the DP. The expected recoveries were then compared with the experimental ones.

The carryover was expressed by the following eq 1 and considered acceptable if <0.1%.

$$\mathrm{carryover}\%=\frac{\mathrm{mRNA}\;\mathrm{area}\;\mathrm{in}\;\mathrm{the}\;\mathrm{blank}\;\mathrm{following}\;\mathrm{DS}\;\mathrm{inj}.}{\mathrm{mRNA}\;\mathrm{area}\;\mathrm{in}\;\mathrm{the}\;\mathrm{DS}}\times 100$$ 1

### Encapsulation Efficiency

The encapsulation efficiency was calculated by dividing the peak area of free mRNA, obtained by injecting undiluted samples, by the peak area of total mRNA obtained after disrupting the LNP structure, according to eq 2.

$$EE=1-\frac{\mathrm{Free}\;\mathrm{mRNA}}{\mathrm{Total}\;\mathrm{mRNA}}=1-\frac{\mathrm{Area}\;\mathrm{undiluted}\;\mathrm{sample}}{\mathrm{Area}\;\mathrm{disrupted}\;\mathrm{sample}\times 10}$$ 2

For LNP disruption, samples were diluted 10 times in TE20X–Triton X-100 4%, and results were compared with those obtained using the TE20X–Triton X-100 reduced 4%. As no differences were observed between the two surfactants, the reduced form of Triton X-100 was selected for further experiments due to its lower environmental impact. The resulting buffer solution is referred to as T4TE20X.

The plots comparing the EE values obtained with RiboGreen and AEX using linear correlation were constructed with GraphPad Prism v10 (GraphPad Software, USA).

## Results and Discussion

### Column Comparison (Carryover, Linearity)

Due to a high number of negative charges and complex structure, mRNA is prone to nonspecific adsorption and strong retention under AEX conditions, leading to significant carryover between analyses, as reported elsewhere.^34^ To estimate the carryover, an injection of 2 μL of DS diluted at 1 mg/mL in water was performed, followed by a blank injection. To mitigate carryover issues and obtain values below 0.1%, the washing procedure was optimized on two strong anion exchanger stationary phases (Figure 1). After eluting mRNA with a 0–1.5 M NaCl gradient at pH 10.1, various washing conditions were tested, including high pH (11.0–11.5) or high NaCl concentration (3–5 M) for times of up to 5 min. As shown in Figure 1, the carryover decreased significantly with longer wash times. However, neither high pH nor high salt alone was sufficient to reduce carryover below 1%. Interestingly, combining pH 11.0 with 3 M NaCl effectively reduced carryover below 0.1% in just 0.5 min. This effect is likely due to Cl^–^ competition for binding sites and increased neutralization of residual functional groups on the stationary phase under alkaline conditions. To ensure wash effectiveness, we implemented a 2 min isocratic step following mRNA elution.

**Figure 1:**
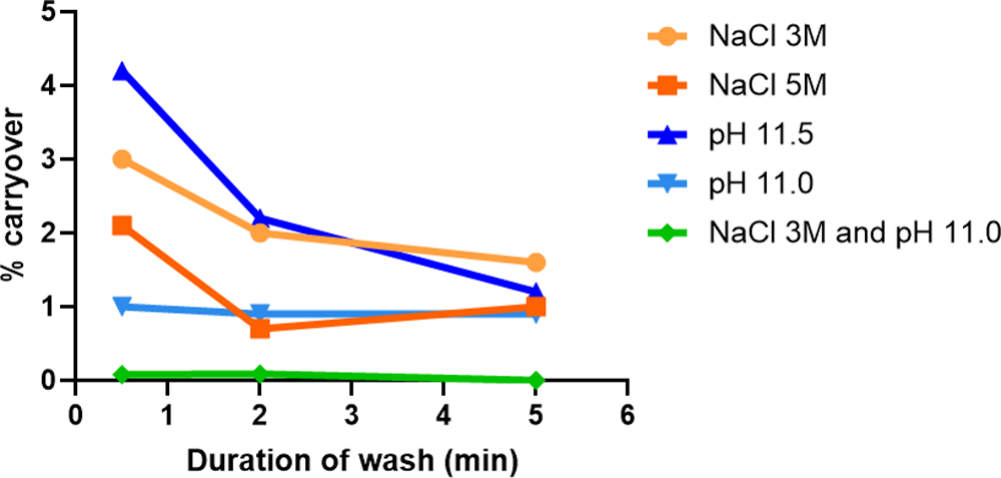
Carryover (%) obtained in the TE20x blank following injection of 1 mg/mL DS, under different conditions of wash composition and duration. Mobile phase A was composed of 25 mM glycine, pH 10.1; and mobile phase B was composed of 25 mM glycine, pH 10.1, 1.5 M NaCl. The flow rate was 0.2 mL/min, and the injected volume was 2 μL. The wash was initially composed of 100% mobile phase B. Its NaCl concentration and pH were optimized to reduce the carryover.

Qualification data were used to choose the optimal AEX stationary phase. For both columns, *y*-intercepts and slopes measured on DS in TE20x were comparable, and *R* ^2^ values were always higher than 0.99 (Figure S1). Accuracy, measured with LNP matrices spiked with DS, showed significant variations, depending on the buffer composition. Indeed, mRNA recovery increased from 20% in pure water to 60% in TE1X, reaching 100% in TE20X (Table 1). Of note, initial tests were performed on a YMC Accura column (rather than an Accura BioPro column), which exhibited significant carryover and poor result reproducibility, highlighting the need for low-adsorption material.

**Table 1:** Recovered mRNA from Diluted DS Spiked in mRNA-LNP Drug Product (DP), Depending on the Buffer Composition^a^

samples	theoretical % free mRNA	experimental % free mRNA	recovery
DS 50% + 50% DP in H20	51	11	22
DS 50% + 50% DP in TE1X	51	32	63
DS 50% + 50% DP in TE20X	54	55	102

a The expected theoretical values are corrected with the free mRNA found in the DP alone.

When columns were compared, a higher variability was observed when using TE20X with the Thermo ProPac 3R SAX recovery (RSD up to >10%) compared to the YMC Accura BioPro IEX QF (RSD always below 2%), as shown in Figure 2 and Table S1. Based on these findings, the YMC column was selected for further method optimization.

**Figure 2:**
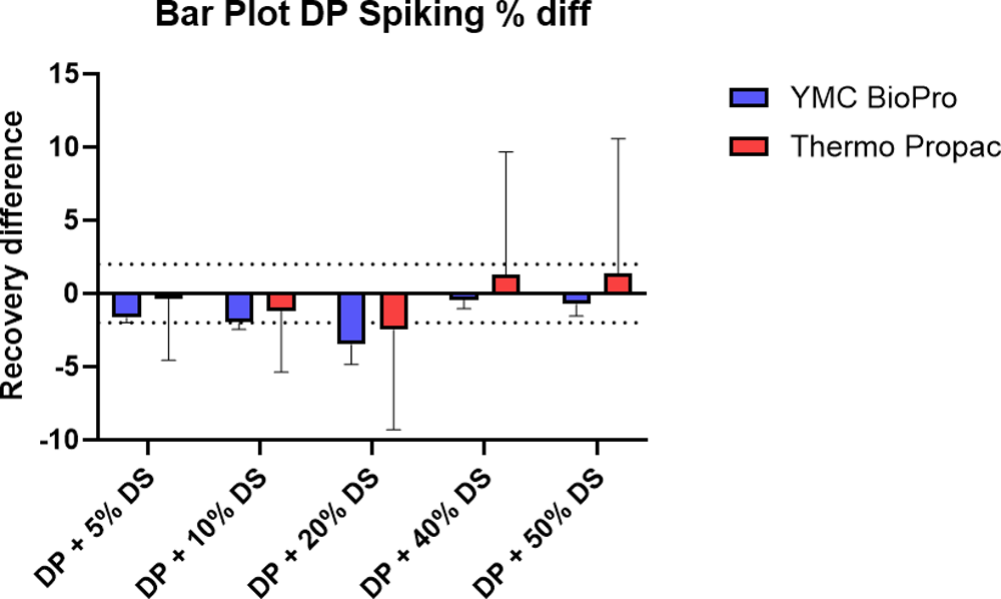
Recovered mRNA from DP samples diluted with an increasing percentage of DS, in the TE20x buffer. Two different columns were used: Thermo ProPac 3R SAX and YMC Accura BioPro IEX QF. Analyses were performed in triplicate. Mobile phase A was composed of 25 mM glycine, pH 10.1; and mobile phase B was composed of 25 mM glycine, pH 10.1, 1.5 M NaCl. The flow rate was 0.2 mL/min, and the injected volume was 2 μL.

### mRNA Carryover Caused by Release from LNP

During method development, a previously unreported form of carryover was identified. It was characterized by the persistent retention of LNPs within the system after DP injection. Indeed, due to their size and physicochemical properties, LNPs tend to adsorb onto surfaces, and the strong washing conditions optimized for mRNA removal were ineffective at eliminating them. Our data indicate that these retained LNPs gradually degrade over time, slowly releasing encapsulated mRNA. Notably, despite an initial blank injection following DP analysis showing no detectable mRNA, a second blank injection was performed several hours later, which confirmed the gradual degradation and release of encapsulated content (Figure S2). Additionally, we observed that a blank injection consisting of T4TE20X and performed between DP injections showed a significant amount of mRNA, supporting an accelerated degradation of retained LNPs related to the surfactant injection (Figure S3). This is an important issue since disrupted samples are diluted in T4TE20X, meaning that any disrupted samples would trigger additional mRNA release, leading to an overestimation of total mRNA.

To eliminate this bias and because the surfactant concentration in a single injection was insufficient to fully disrupt all retained LNPs, we incorporated an amount of 0.05% Triton X-100 reduced into mobile phase C. This additional wash effectively removed all retained LNPs and reduced carryover to undetectable levels, even in blanks composed of T4TE20x (Figure S4). To the best of our knowledge, in-column mRNA release from retained LNPs has not been previously reported. These findings highlight the need for careful assessment of LNP retention in chromatographic method development, particularly when using AEX.

### Surface-Associated mRNA

Interestingly, a distinct pre-mRNA peak was observed in several DP samples. This additional species absorbed across all UV wavelengths and had a retention close to the mRNA main peak, suggesting a large UV-absorbing entity with an overall negative surface charge. To assess potential light-scattering effects in UV, we evaluated the 260/230 nm absorbance ratio. Its value was always close to 1, supporting the hypothesis of an mRNA-LNP association. Notably, this peak was absent in both empty LNP formulations and empty LNPs spiked with mRNA at neutral pH. However, it was observed in empty LNPs spiked with mRNA under acidic pH, where ionic lipids are positively charged, indicating that electrostatic interactions at low pH may promote the formation of these complexes (Figure 3).

**Figure 3:**
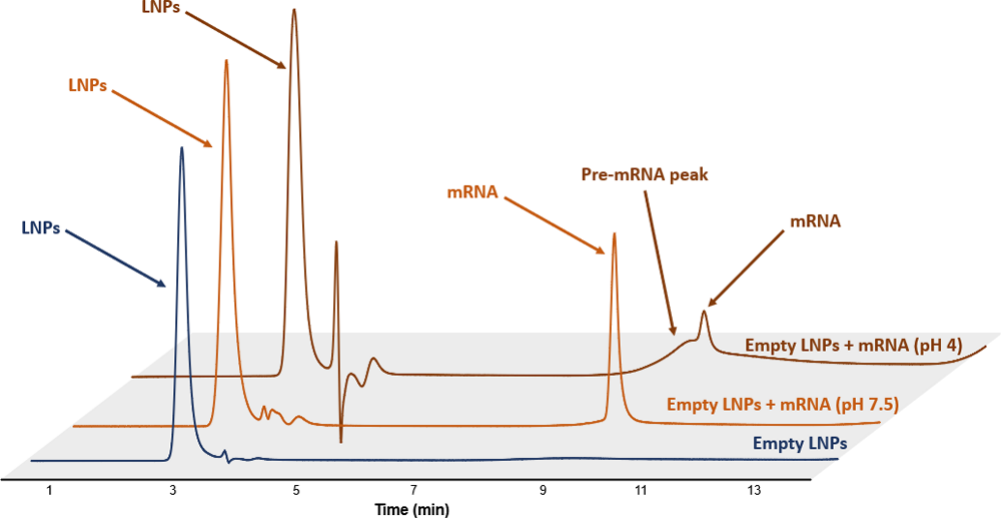
Chromatographic profiles of empty LNPs spiked with mRNA at two different pH conditions: pH 4 and pH 7.5. Peaks of mRNA are observed in all conditions, while pre-mRNA peaks are only visible at low pH. Mobile phase A was composed of 25 mM glycine, pH 10.1; and mobile phase B was composed of 25 mM glycine, pH 10.1, 1.5 M NaCl. The flow rate was 0.2 mL/min, and the injected volume was 2 μL.

Moreover, the peak was present in water-diluted mRNA-LNPs, but not in TE20X-diluted mRNA-LNPs at pH 7.5 (Figure 4). Again, these findings suggest that pH plays a major role in preventing LNP-mRNA interactions that lead to the presence of surface mRNA. Interestingly, for samples already stabilized using buffers, dilution in TE20X did not remove pre-existing surface mRNA, due to strong interactions between the mRNA molecules and the LNP surface, which may highlight the presence of transmembrane mRNA.^38^

**Figure 4:**
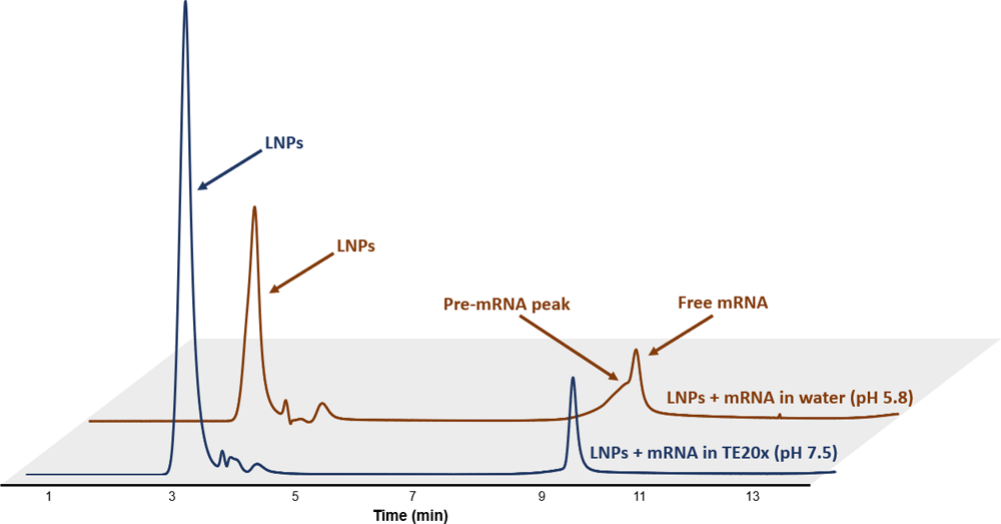
Chromatographic profiles of drug products diluted in water or TE20x buffer. Free mRNA peaks are observed in all conditions, while the pre-mRNA peak is only visible in the water condition. Mobile phase A was composed of 25 mM glycine, pH 10.1; and mobile phase B was composed of 25 mM glycine, pH 10.1, 1.5 M NaCl. The flow rate was 0.2 mL/min, and the injected volume was 2 μL.

Finally, the peak was not observed systematically in all samples and is generally increasing over time. All of these observations confirm the hypothesis of surface-associated mRNA-LNP.

To ensure that the pre-mRNA peak did not alter the measurement of mRNA due to light-scattering effects observed with UV detection, an isocratic step was introduced between the two species to clearly differentiate them. Because large molecules such as mRNA follow an on/off retention mechanism, this strategy does not result in peak broadening for the peak eluted after the isocratic step.^39^ The gradient was fine-tuned to improve resolution while maintaining suitable peak shapes and avoiding mRNA peak splitting, as illustrated in Figure 5.

**Figure 5:**
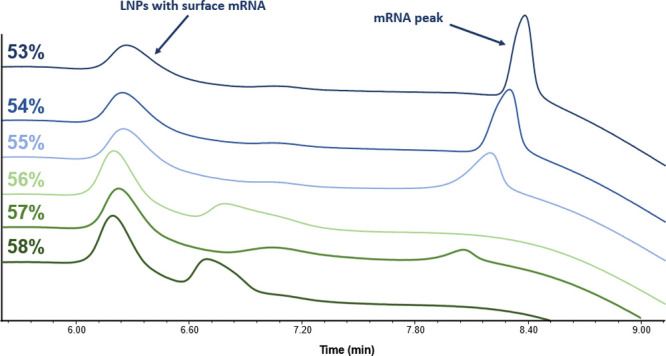
Chromatographic profile of the optimization of the isocratic step between the mRNA main peak and the pre-mRNA peak. Mobile phase A was composed of 25 mM glycine, pH 10.1; and mobile phase B was composed of 25 mM glycine, pH 10.1, 1.5 M NaCl. The flow rate was 0.2 mL/min, and the injected volume was 2 μL. The step was 1.75 min long and set at 53–58% mobile phase B. The isocratic step at 53% was kept in the final method.

Besides allowing the successful calculation of EE, our AEX method offers the first experimental evidence of surface-localized mRNA within LNPs and the probable presence of transmembrane mRNA. Because of light-scattering effects, the pre-mRNA peak cannot be quantified accurately, but its presence provides qualitative structural information about mRNA distribution within the LNPs. This information could support future method developments and formulation strategies. Interestingly, in some cases, this peak appears to be stability-indicating, since its area increases with storage time. However, it should not be considered as a direct degradation marker but rather a structural indication, as it is also present in freshly prepared samples.

### Encapsulation of Drug Products and Comparison with RiboGreen Results

The final AEX method for EE assessment was the following: mobile phase A consisted of 25 mM glycine, pH 10.1, while mobile phase B contained 25 mM glycine, pH 10.1, and 1.5 M NaCl. To remove retained LNPs and eliminate carryover, mobile phase C was formulated with 25 mM glycine, pH 11.0, 3 M NaCl, and 0.05% Triton X-100 reduced. The analysis was performed at 25 °C with a flow rate of 0.2 mL/min to limit pressure drop and shear forces, and the optimal gradient conditions including the isocratic step are detailed in Table S2. Thanks to these conditions, we were able to stay below a pressure of 1000 psi, thereby avoiding disruption of the LNPs.

The repeatability of the method was assessed by injecting the same undiluted DP sample 50 times consecutively, each injection followed by the corresponding disrupted DP sample, and calculating EE for each pair (Figure S5). As shown in Table S3, the method demonstrated excellent repeatability, with an RSD value of only 0.28%.

Then, the method was applied to the analysis of 30 different mRNA-based vaccine candidates currently under development. These included both single- and multipayload formulations, with mRNA lengths ranging from 1000 to 2500 nucleotides, encapsulated in lipids of varying composition. EE was determined in triplicate using our AEX method, and the results were systematically compared to those obtained with the RiboGreen assay.

As shown in Figure 6, EE values measured with AEX and the RiboGreen assay exhibited a moderate positive correlation (*R* ^2^ = 0.67, *p* < 0.0001). This result suggests that AEX and RiboGreen highlight similar trends in encapsulation levels across samples, but external factors generate different results, which are likely related to the mRNA accessibility to the RiboGreen dye. Indeed, the permeability of the LNP membrane and the surface mRNA are affected by sample type and buffers, which should explain the observed differences. Moreover, except for one sample, EE measured by AEX was always higher than EE measured with RiboGreen. Again, this supports the idea that RiboGreen interacts with species beyond purely dissociated mRNA from LNPs.

**Figure 6:**
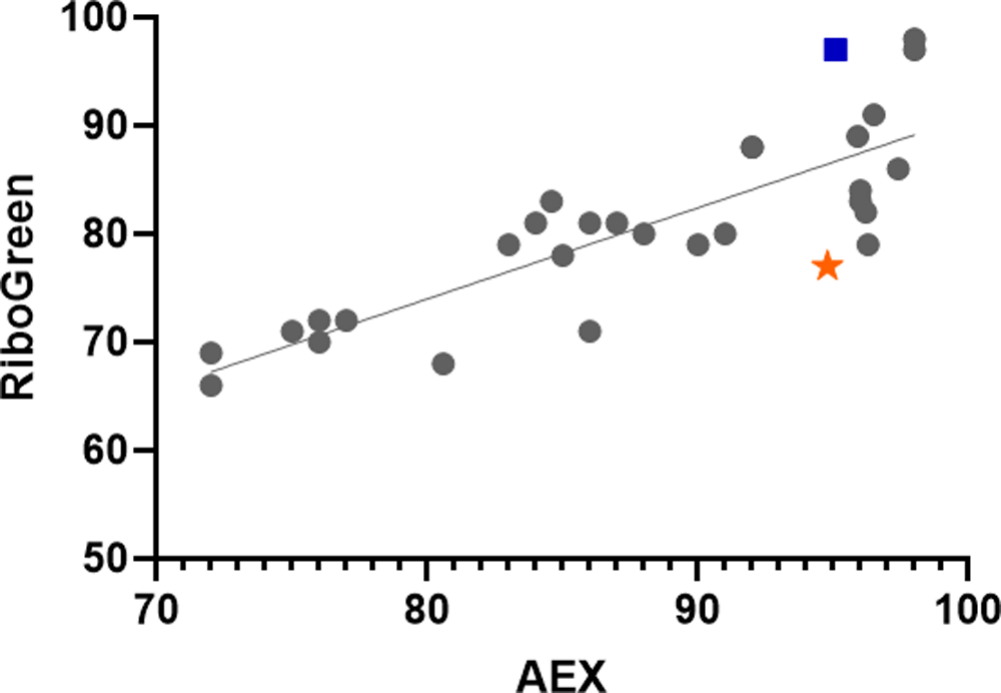
Correlation between EE values obtained with the RiboGreen assay and the AEX method on 30 samples. Samples in blue square and orange star are further described in the text.

To better understand the discrepancies observed between the two methods, we further explored the chromatograms obtained. As an illustration, we took the two samples highlighted in Figure 6. The blue square represents a sample with a high EE of 95.1 and 97.0%, obtained with AEX and RiboGreen, respectively. For this sample, both EE values were extremely close, and the RiboGreen assay gave a result slightly above the one obtained with AEX. Interestingly, the AEX chromatogram obtained for this sample, presented in Figure 7 (blue trace), did not show any peak associated with surface mRNA. In this case, no structural particularity was observed by the AEX method, and the two assays gave similar results. On the other hand, the orange star in Figure 6 represents a sample with an important difference in EE values obtained with the two techniques. EE values were 94.8 and 77.0% for AEX and RiboGreen, respectively. For this sample, the AEX chromatogram (brown trace in Figure 7) exhibited an intense peak associated with on-surface mRNA. Due to light-scattering effects, integration of the peak does not provide reliable quantitative data, but its presence serves as an indicator of surface mRNA-LNP complex formation.

**Figure 7:**
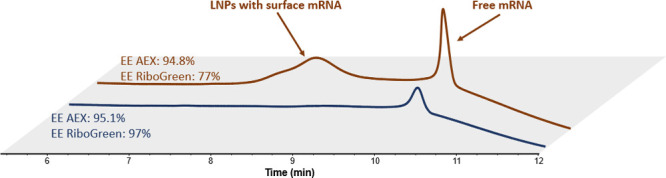
Chromatograms obtained for the samples highlighted in Figure 6, with the corresponding colors. Encapsulation efficiencies obtained with the RiboGreen assay and AEX method are expressed in %. Main mRNA peak and surface-bounded mRNA are highlighted. Mobile phase A was composed of 25 mM glycine, pH 10.1; mobile phase B was composed of 25 mM glycine, pH 10.1, 1.5 M NaCl, and mobile phase C was composed of 25 mM glycine, pH 11.0, NaCl 3M and Triton X-100 reduced 0.05% v/v. The flow rate was 0.2 mL/min, and the injected volume was 2 μL.

Based on the hypothesis that this pre-mRNA peak corresponds to LNPs with surface-exposed mRNA, our findings suggest that the RiboGreen assay considers surface and/or transmembrane mRNA as unencapsulated. This behavior is due to the partial accessibility of surface mRNA to the RiboGreen dye. Thus, the RiboGreen method gives a percentage of mRNA accessible to the fluorescent dye, which could eventually lead to free mRNA overestimation, especially if some forms of LNPs have some permeability to the dye or express surface-associated or transmembrane mRNA. Therefore, our AEX method is not only simpler, faster, more cost-effective and repeatable than RiboGreen, but it also provides crucial structural information about the mRNA-LNP drug product. Compared to prior reports on anion exchange for EE evaluation,^36^ our method used real drug product samples, thoroughly investigates mRNA carryover and mRNA-LNP interactions across buffers, and reveals for the first time the presence of surface-bound mRNA. Thus, it offers an accurate evaluation of truly free mRNA and serves as a powerful tool for understanding formulation differences, structural changes, and encapsulation characteristics in an industrial context.

These results finally raise the question of which information is given by the encapsulation rates. In our opinion, encapsulation efficiency should reflect the amount of mRNA that would reach the cytoplasm and be effectively translated into protein. Therefore, further studies are needed to evaluate if transmembrane and surface-associated mRNA can still be translated into proteins and to understand which analytical technique most accurately correlates with protein expression. If discrepancies are observed between RiboGreen-based EE values and actual protein expression, they could be explained by RiboGreen inability to differentiate free and surface-associated mRNA. Additional expression studies and complementary characterization techniques should be developed to distinguish surface-associated mRNA, transmembrane-bound mRNA, and fully LNP-encapsulated mRNA, ultimately determining whether the AEX method provides a more accurate and biologically relevant measure of encapsulation efficiency.

## Conclusions

In this work, we developed an AEX platform method to evaluate the encapsulation efficiency (EE) of mRNA-LNP therapeutics and vaccines. The method involves two injections: the first injection measures free mRNA in an intact drug product, while the second, after offline disruption, quantifies total mRNA.

To ensure the reliability of EE measurements, we thoroughly investigated mRNA carryover and identified two distinct mechanisms. The first arises from the residual mRNA drug substance retained within the chromatographic system, while the second, previously unreported, resulted from the delayed release of mRNA from LNPs adsorbed to the system. Both carryover effects were effectively reduced to undetected levels by optimizing the wash protocol, specifically by increasing salt concentration and pH and incorporating 0.05% v/v Triton X-100 reduced in the wash solution.

During method development, we detected an additional peak in certain samples, characterized by light scattering under UV detection and with a retention time close to that of mRNA. Our data suggest that this peak corresponds to LNPs containing surface-bound or transmembrane mRNA. Its presence was influenced by several factors, such as sample type, buffer composition, and storage time. To minimize the impact of light-scattering effects on the mRNA peak, which could lead to inaccurate EE estimation, we introduced an isocratic step to achieve better separation of these species. This adjustment not only improved the analysis of mRNA encapsulation but also provided structural insights into mRNA-LNP complexes.

The method was then applied to 30 DP samples, and results were compared with those of the RiboGreen assay, the current reference for EE measurement. Although the two methods exhibited a moderately positive correlation, notable differences emerged due to structural variations affecting mRNA accessibility to the RiboGreen dye. Notably, the AEX and RiboGreen results aligned well for samples without surface mRNA, whereas samples with high surface mRNA showed significant discrepancies, highlighting the hypothesis that the RiboGreen assay detects surface-associated mRNA as unencapsulated.

In conclusion, this fit-for-purpose method provides novel insights into LNP surface-bound and transmembrane mRNA, contributing to a better understanding of structural changes and stability. Compared to previously reported assays for EE evaluation, this work carefully evaluated carryover issues to improve the reliability of the results and LNPs-mRNA interactions and used real drug products. Our findings highlight the need for more precise analytical tools in mRNA drug product characterization. Further studies are necessary to assess the biological impact of surface and transmembrane mRNA and confirm whether AEX-based EE measurements more accurately reflect functional mRNA delivery and translation. By offering a more comprehensive and reliable analytical approach, AEX has the potential to serve as an orthogonal method for batch release and quality control of mRNA-LNP formulations.

## Supplementary Material



## Notes

Prequalification data, mRNA recovered, description of the gradient, encapsulation efficiency, AEX gradient details, method EE RSD, and carryover issues (PDF)
