# Supplementary material for: Anion Exchange Chromatography to Determine mRNA Encapsulation in Lipid Nanoparticles
Source: Anal Chem. 2025 Aug 22;97(35):19275–82. doi: 10.1021/acs.analchem.5c03299 (PMC12424022; doi:10.1021/acs.analchem.5c03299)
Supplement: Supplementary file 1 [file ac5c03299_si_001.pdf]

SUPPORTING INFORMATION

Anion exchange chromatography to determine mRNA encapsulation in lipid nanoparticles

Athanasios Tsalmpouris[1,2$], Sofiane Mahjoubi[1,2$], Camille Malburet[3], Chamsan Daher Hassan[3], Marc François-Heude[3], Jean-François Cotte[3], Davy Guillarme*[1,2], and Jonathan Maurer[1,2,3]

[1] School of Pharmaceutical Sciences, University of Geneva, CMU-Rue Michel Servet 1, 1211 Geneva, Switzerland.

[2] Institute of Pharmaceutical Sciences of Western Switzerland, University of Geneva, CMU-Rue Michel Servet 1, 1211 Geneva, Switzerland.

[3] mRNA Center of Excellence, Analytical Sciences, Sanofi, 1541 Avenue Marcel Mérieux, 69280 Marcy l'Etoile, France.

$ A.T. and S.M. contributed equally to this paper

Table of Content

Figure S1. Prequalification data for the two columns 2

Figure S2. mRNA recovered in a blank after >15h 3

Figure S3. mRNA recovered in a T4TE20x blanks 3

Figure S4. mRNA recovered in a T4TE20x blank with optimized wash step 4

Figure S5. Overlay of the chromatograms obtained for repeatability measurements 4

Table S1. Recovered mRNA from a DP spiked with DS, for the two columns 5

Table S2. LC gradient used in the optimized method 5

Table S3. Encapsulation efficiencies measured 50 times on a sample.......... 6

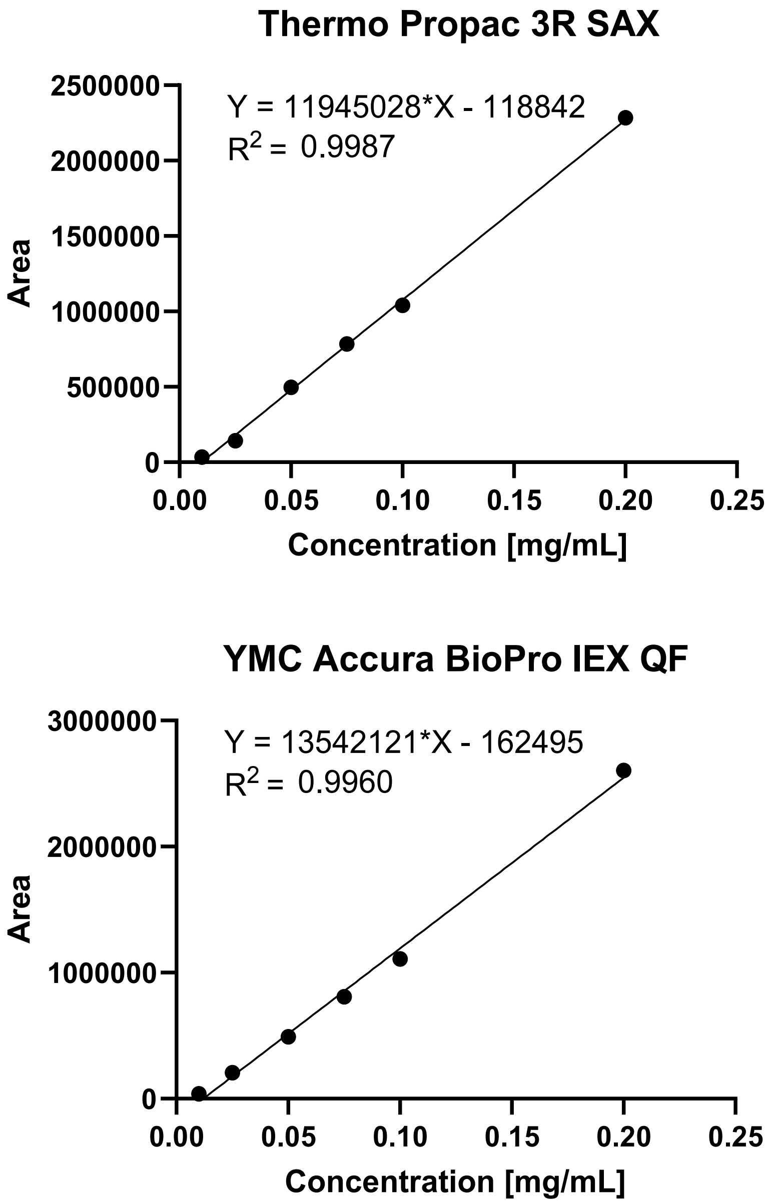

Figure S1. *Prequalification data for the ProPac 3R SAX 4 x 100 mm, 3 µm column from Thermo Fischer and the Accura BioPro IEX QF 4.6 x 100 mm, 3 µm from YMC. Calibration curves of the DS diluted in TE20x in 6 different concentration levels show a strong linear relationship for both columns.*

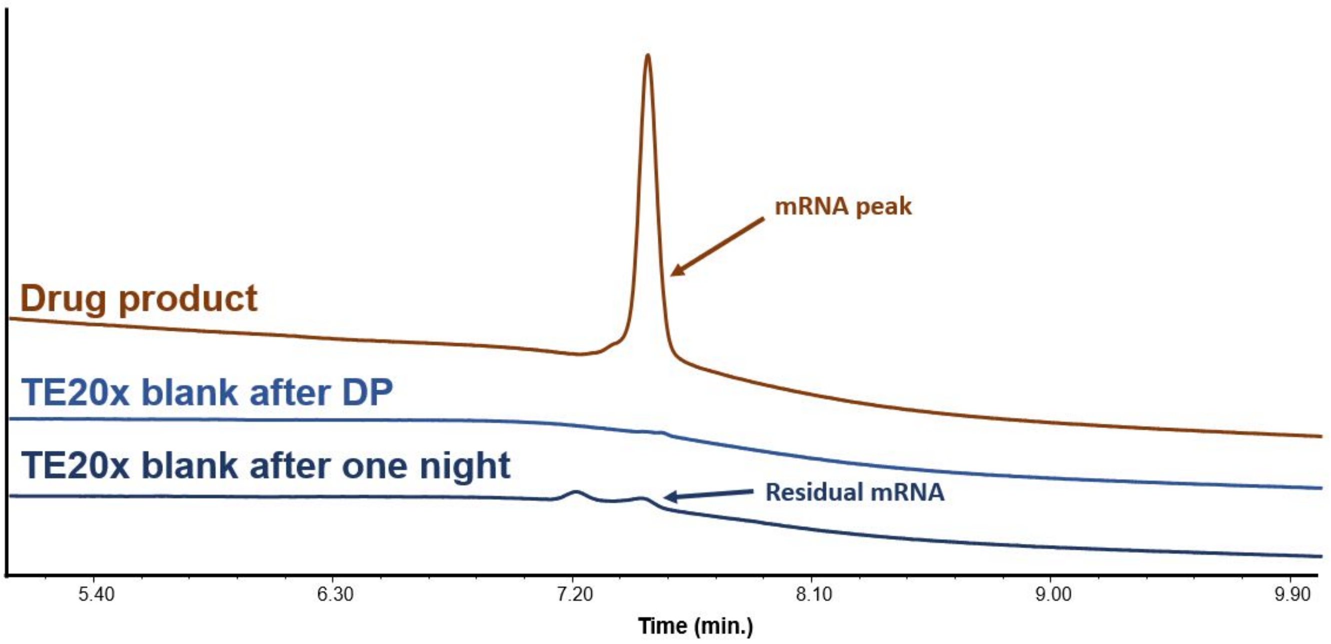

Figure S2. *mRNA recovered from the injection of a drug product, followed by two TE20x blanks. One was performed right after DP injection, and the other was performed the after >15h. No mRNA was observed in the first blank, while the other showed significant amounts, supporting a slow degradation of retained LNPs in the system resulting in a release of mRNA.*

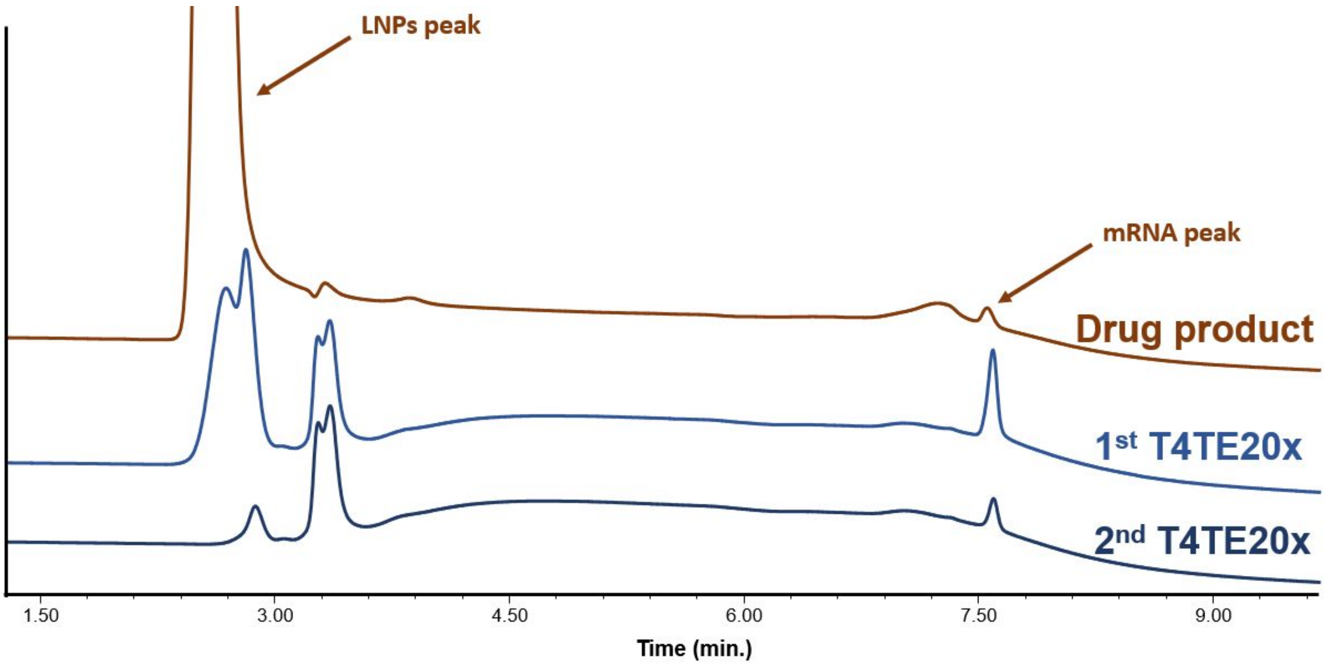

Figure S3. *mRNA recovered from the injection of a drug product, followed by two T4TE20x blanks. The LNP peak on the left is slowly decreasing from one injection to another, and significant amount of mRNA is recovered in the blanks following DP injection. This supports the disruption of retained LNPs by Triton X-100 reduced injections. However, even after two T4TE20x blanks, significant mRNA is still recovered, highlighting the need of optimized wash steps.*

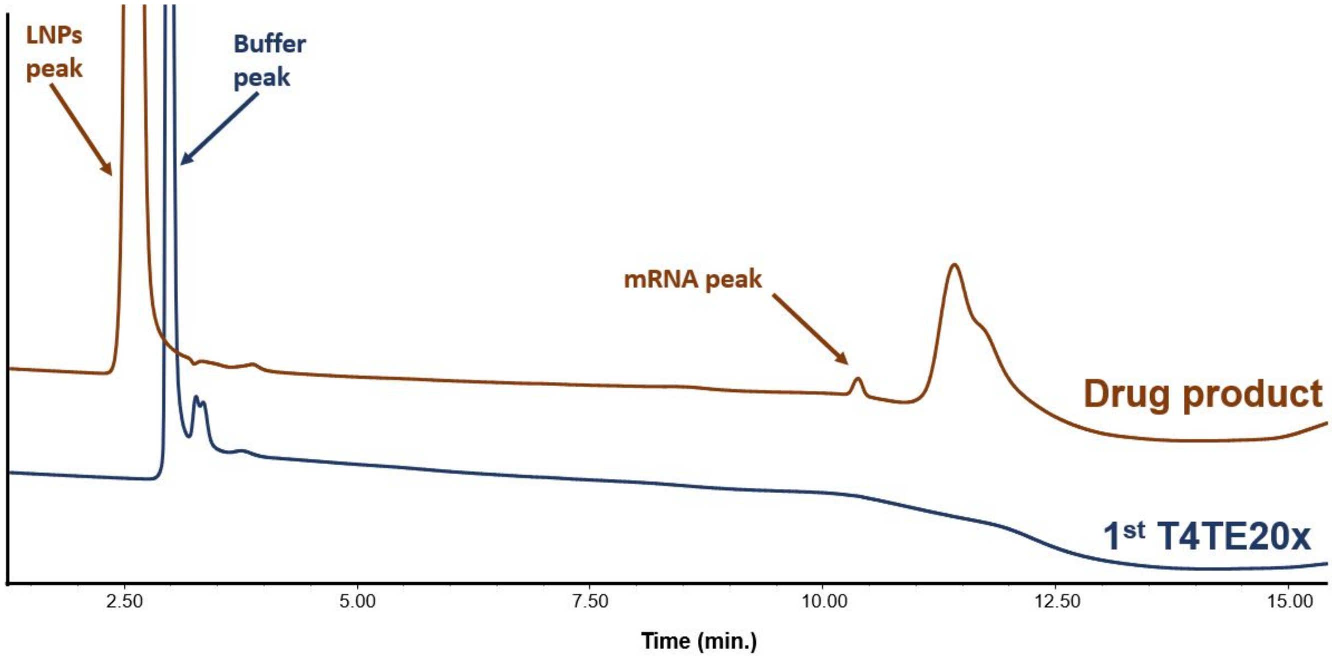

Figure S4. *mRNA recovered from the injection of a drug product, followed by a T4TE20x blank, using the optimized method with a wash containing 0.05% Triton X-100 reduced. The following T4TE20x showed no mRNA, confirming the effectiveness of the wash to remove retained LNPs.*

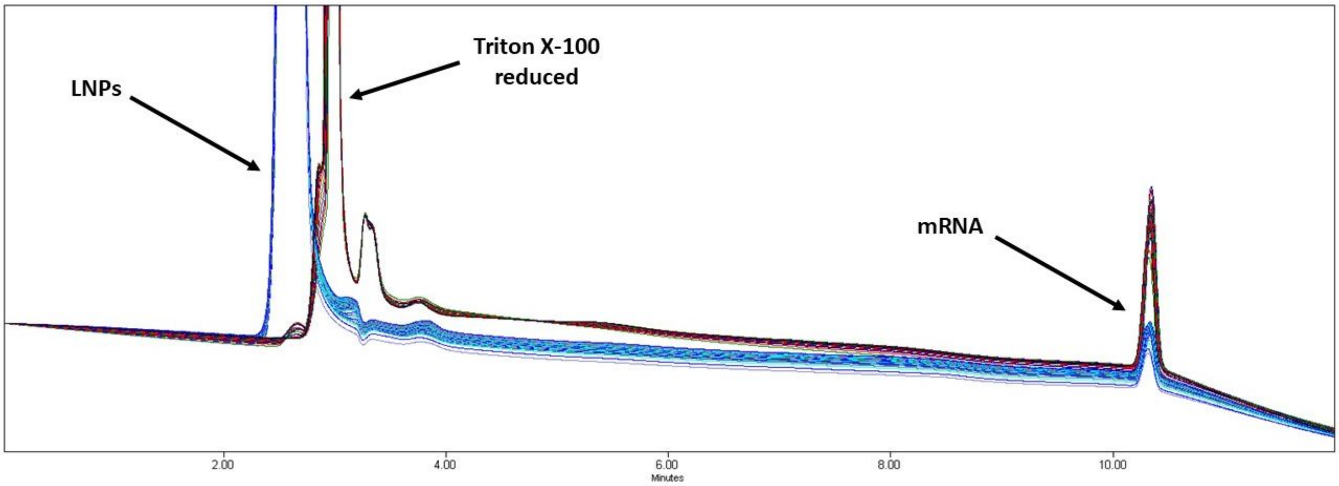

Figure S5. *Overlay of the 50 intact DP injections and the 50 corresponding disrupted DP samples diluted in Triton X-100 reduced.*

Table S1. *Recovered mRNA from diluted drug substance (DS) in drug product (DP) composed of mRNA-LNP vaccines. Both DP and DS are diluted in TE20x.*

	**YMC Accura Biopro**		**Thermo ProPac 3R SAX**	
	Experimental % Free mRNA	**Delta**	Experimental % Free mRNA	**Delta**
100% DP + 0% DS	0.2%		0.7%	
95% DP + 5% DS	3.7%	- 1.3	3.7%	- 1.3
90% DP + 10% DS	8.5%	- 1.5	9.1%	- 0.9
80% DP + 20% DS	18.0%	- 2	17.3%	- 2.7
60% DP + 40% DS	40.2%	+ 0.2	44.0%	+ 4
50% DP + 50% DS	50.0%	0	55.1%	+ 5.1

Table S2. *Description of the gradient used in the optimized AEX method, for mobile phases A, B, and C.*

**Time (min.)**	**%A: Glycine 25 mM – pH 10.1**	**%B: Glycine 25 mM – pH 10.1 – NaCl 1.5 M**	**%C: Glycine 25 mM – pH 11 – NaCl 3 M – 0.05% Triton X-100 reduced**
0	50	50	0
2	50	50	0
2.25	47	53	0
4	47	53	0
6	0	100	0
8	0	100	0
8.01	0	0	100
12	0	0	100
12.01	50	50	0
22	50	50	0

Table S3. *Encapsulation efficiency obtained for an undiluted sample and disrupted sample, injected 50 times in a row with the optimized AEX method.*

**Injection**	**Encapsulation efficiency [%]**	**Injection**	**Encapsulation efficiency [%]**
1	96.94	26	96.71
2	97.03	27	96.63
3	97.17	28	96.65
4	97.24	29	96.65
5	97.51	30	96.68
6	97.58	31	96.67
7	97.53	32	96.80
8	97.45	33	96.70
9	97.29	34	96.67
10	97.15	35	96.65
11	97.17	36	96.63
12	97.14	37	96.62
13	96.85	38	96.57
14	96.78	39	96.66
15	96.70	40	96.65
16	96.69	41	96.60
17	96.68	42	96.76
18	96.70	43	96.68
19	96.71	44	96.68
20	96.76	45	96.76
21	96.71	46	96.73
22	96.92	47	96.73
23	96.75	48	96.64
24	96.87	49	96.62
25	96.78	50	96.61
**RSD**		**0.28%**	
